# Contribution of Phenolic Acid Profiles to the Anti-Adipogenic Activity of Different *Mesona procumbens* Hemsl. Ethanol Extracts

**DOI:** 10.3390/biomedicines14040824

**Published:** 2026-04-04

**Authors:** Ching-Chang Cho, Gow-Chin Yen, Hsin-Yi Lee, Wei-Tang Chang, Li-You Chen, Chin-Lin Hsu

**Affiliations:** 1 Department of Nutrition, Chung Shan Medical University, Taichung 40201, Taiwan; ccjwo21@gmail.com (C.-C.C.); huonede@gmail.com (H.-Y.L.); 2 Department of Food Science and Biotechnology, National Chung Hsing University, Taichung 40227, Taiwan; gcyen@nchu.edu.tw; 3 Department of Nutrition and Health Sciences, Chinese Culture University, Taipei 11114, Taiwan; zwt6@ulive.pccu.edu.tw; 4 Department of Anatomy, School of Medicine, College of Medicine, Chung Shan Medical University, Taichung 40201, Taiwan; peiyu@csmu.edu.tw; 5 Department of Nutrition, Chung Shan Medical University Hospital, Taichung 40201, Taiwan

**Keywords:** anti-lipid accumulation, antioxidant, phenolic acid, Hsian-tsao, *Mesona procumbens* Hemsl., 3T3-L1 adipocytes, obesity

## Abstract

**Background/Objectives**: Obesity represents a critical risk factor for various chronic illnesses and metabolic dysfunctions, underscoring the urgency of identifying safe, food-based interventions to curb fat over-accumulation. *Mesona procumbens* Hemsl. (Hsian-tsao) is a traditional Chinese herb known for its antioxidant and health-promoting properties; however, it remains unclear how its phenolic acid profiles contribute to anti-obesity activity. This research explored the anti-adipogenic potential of various Hsian-tsao ethanol extracts, focusing on how their phenolic profiles influence lipid suppression in 3T3-L1 adipocytes. **Methods**: Ethanol extracts prepared using different ethanol concentrations were analyzed for total polyphenol content, antioxidant capacity, and phenolic acid profiles. Adipocytes were exposed to 0, 100, and 250 μg/mL of Hsian-tsao ethanol extract for 48 h duration to monitor changes in cell count and intracellular triglyceride levels. **Results**: Among all fractions, the 40% ethanol extract (40EEHT) possessed the most robust antioxidant capacity and highest polyphenol content, specifically showing enriched levels of caffeic acid, *p*-coumaric acid, and total phenolic acids. Notably, while 40EEHT influenced cell density at certain concentrations, it significantly and specifically reduced intracellular triglyceride content, indicating a potent inhibitory effect on lipid storage independent of changes in cell number. Comparative analysis using phenolic acid standards revealed that caffeic acid exerted the strongest inhibitory effect on lipid accumulation, suggesting that it is a key contributor to the anti-adipogenic activity of 40EEHT. **Conclusions**: Collectively, these findings demonstrate that phenolic acid profiles, particularly caffeic acid enrichment, critically contribute to the potential anti-adipogenic effects of specific ethanol extracts of *M. procumbens*. Therefore, Hsian-tsao ethanol extracts represent a promising natural source for the development of functional ingredients targeting obesity and related metabolic disorders.

## 1. Introduction

Obesity has become a major metabolic disorder worldwide [1] and is related to various other syndromes, including hypertension, coronary artery disease, respiratory syndromes, type II diabetes mellitus, osteoarthritis, and cancer [2]. It is a progressive condition characterized by fat accumulation and expanded adipose cell size [3]. Common anti-obesity medications used in treatment include setmelanotide, phentermine/topiramate, liraglutide, orlistat, semaglutide, and bupropion/naltrexone. However, these medications may cause side effects such as decreased fat-soluble vitamin absorption, flatulence, oily stool, and diarrhea [4,5]. Therefore, several anti-obesity food science and technology investigations have focused on exploring various bioactive compounds with anti-obesity potential [6,7,8].

Chinese herbs are known to have various functional components and are often used as active ingredients in traditional Chinese medicine [9,10]. Hsian-tsao (*Mesona procumbens* Hemsl.) is an edible and medicinal plant widely used in traditional herbal medicine to ameliorate muscle pain, hypertension, liver disease, heat shock, and diabetes [11]. Previous studies have indicated that Hsian-tsao displays anti-oxidative, anti-inflammatory, anti-mutagenic, and liver- and kidney-protective effects [11,12,13,14].

Furthermore, Hsian-tsao is rich in phenolic acids, including chlorogenic acid (CGA), protocatechuic acid (PCA), *p*-hydroxybenzoic acid (*p*-HBA), caffeic acid (CA), vanillic acid (VA), and *p*-coumaric acid (*p*-CA) [12,15]. Numerous investigations have shown that these six phenolic acids can ameliorate obesity and related symptoms. In recent studies, PCA has been shown to improve high-fat-diet-induced insulin resistance and obesity by activating fatty acid oxidation and the browning of white adipose tissue [16]. It also significantly improved body weight and body fat and blocked metabolic endotoxemia in HFD-induced obese mice via the intestinal microbiota [17]. *p*-HBA from *Maclura tricuspidata* (Carr.) Bur fruit extract could act as a vital factor in glucose and lipid metabolism by inhibiting enzymes associated with the pathogenesis of obesity, such as pancreatic lipase, α-amylase, and β-glucosidase [18]. In an in vivo study, HFD-induced obese mice that received 50 mg/kg of CA per day for 12 weeks showed a marked reduction in body weight and lipid accumulation. CA also promoted beneficial gut bacteria, including *Muribaculaceae*, and reduced pathogenic bacteria, including *Lachnospiraceae*, in the gut microbiota [19]. Vanillic acid and other biofunctional compounds (like β-glucan and ferulic acid) in *Lactobacillus plantarum* dy-1-fermented barley extracts (LFBEs) markedly relieved body weight, glucose tolerance, and lipid contents and decreased fat accumulation in 3T3-L1 adipocytes in high-fat-diet-induced rats [20]. Nathu et al. (2021) indicated that in vitro gastroduodenal digestion of mageu induced the extraction of phenolic acids such as ferulic acid, *p*-coumric acid, caffeic acid, and 3,4-dihydroxybenzoic acid, which increased cellular antioxidant activity and reduced lipid accumulation in 3T3-L1 adipose cells [21].

Since obesity increases the risk of numerous metabolic syndromes, bioactive compounds that may be beneficial in reducing excessive lipid accumulation are worth exploring. Furthermore, 3T3-L1 adipocytes are a useful and well-established in vitro model for obesity or related diseases [22,23]. There is presently no research associated with the beneficial effects of phenolic acids in Hsian-tsao on the modulation of lipogenesis or anti-obesity.

## 2. Materials and Methods

### 2.1. Extraction Process of Hsian-Tsao Using Ethanol

The Mesona procumbens Hemsley (Taoyuan No. 1) used in this study was sourced from the Guanxi Town Farmers Association in Hsinchu County, Taiwan. The raw material of *M. procumbens* Hemsl. was prepared as follows: the dried herb was lyophilized and subsequently ground into a powder using a laboratory blender (Waring 8011S, Waring Commercial, Stamford, CT, USA). This processed powder was then used for the various ethanol extraction procedures described below. To prepare the extracts, 4 g of dried Hsian-tsao powder was subjected to solvent extraction using a variety of ratios of ethanol solution (0–100%, *v*/*v*). The maceration extraction was performed at a ratio of 1:10 (*w*/*v*). To ensure maximal yield of phenolic compounds, a magnetic stirrer (IKA C-MAG HS 7, IKA-Werke GmbH & Co. KG, Staufen, Germany) was used to continuously stir the mixtures for 24 h at room temperature (25 ± 2 °C). Post-extraction, the resultant mixtures underwent centrifugation (3000 rpm,15 min, 4 °C). A rotary evaporator (Büchi Rotavapor R-300, BÜCHI Labortechnik AG, Flawil, Switzerland) was used to filter and concentrate the resulting supernatant under reduced pressure at 40 °C. The concentrates were lyophilized to acquire the final ethanol extracts (EEHT) in powder form. The yield of each extract was calculated and stored at −20 °C until further analysis. The extraction efficiency was determined by the weight ratio of the obtained EEHT to the initial dried powder, as shown in the following equation:

Yield (%) = (weight of EEHT/weight of starting powder) × 100%.

For cell-based experiments, 100% dimethyl sulfoxide (DMSO) was used to resolve the lyophilized EEHT powders to generate stock samples, which were subsequently diluted in the culture medium to ensure the final concentration of DMSO remained under 0.1% (*v*/*v*), thus avoiding cytotoxicity.

### 2.2. Quantification of Total Phenolic Content (TPC)

The concentration of total phenolics in the various EEHT fractions was determined using a colorimetric approach based on the Folin–Ciocalteau method, with gallic acid as the standard for calibration [24]. To initiate the assay, 5 mg of lyophilized extract was reconstituted in 5 mL of deionized water. A 100 μL sample volume was then combined with 200 μL of 2% Na_2_CO_3_ solution. After a 2 min stabilization period, 0.1 mL of 50% Folin–Ciocalteau reagent was introduced into the mixture. Following a 30 min incubation period under dark conditions to allow for full color development, the mixture solutions were determined via a FLUOstar galaxy spectrophotometer (BMG Labtechnologies Ltd., Ortenberg, Germany) at 750 nm. The final TPC values were expressed as milligrams of gallic acid equivalents (GAEs) per gram of dry sample weight.

### 2.3. Assessment of Antioxidant Potential (TEAC Assay)

The methodology of Trolox equivalent antioxidant capacity (TEAC) was evaluated according to the protocol outlined by Arts et al. (2004) [25]. The ABTS·+ radical cation was generated by reacting a 7 mM ABTS solution with 2.45 mM potassium persulfate in a dark environment at room temperature for a duration of 12–16 h. Before the radical scavenging assay, the resulting mixture was carefully adjusted using phosphate-buffered saline (pH 7.4) until a stable absorbance of 0.70 ± 0.02 at 734 nm was reached. For the measurement, 1.5 mL of diluted ABTS·+ solution was mixed with 25 μL of each EEHT extract (or Trolox standard). The mixture was reacted in the dark condition for 6 min and subsequently measured at OD_734_ using a Hitachi U-3000 UV–Vis spectrophotometer (Hitachi, Ltd., Tokyo, Japan). Scavenging activity was established by the reduction in absorbance and is expressed as TEAC values (μmol/g extract), relative to a Trolox standard curve.

### 2.4. Quantification of Phenolic Acid Components

The phenolic acid profile of EEHT was determined via high-performance liquid chromatography (Hitachi, Tokyo, Japan) following the methodology outlined by Kim et al. (2008) [26]. A 2 g sample of dried Hsian-tsao powder was extracted with 10 mL of acetonitrile (containing 0.1 N HCl) and stirred for 2 h at room temperature. A volume of 20 μL of the resulting extract was loaded onto the HPLC system. A LiChrosorb 100 RP-18 column (5 μm, 250 mm × 4.6 mm i.d., E. Merck, Darmstadt, Germany) maintained at 30 °C was used to achieve separation. (A) 0.1% (*v*/*v*) phosphoric acid in distilled H_2_O and (B) 100% acetonitrile constitute the mobile phase. A gradient elution profile was implemented at 1.3 mL/min as follows: 0–5 min, maintained at 10% B; 5–25 min, escalated from 10% to 40% B; 25–35 min, adjusted from 40% to 60% B. The system was finally restored to 10% B (35–40 min) to ensure column re-equilibration. Detection was performed at an absorbance of 280 nm. Standard curves were established using phenolic acid standards (PCA, CGA, *p*-HBA, CA, VA, and *p*-CA; Sigma-Aldrich, St. Louis, MO, USA) prepared in dimethyl sulfoxide (DMSO) at concentrations of 50, 100, 250, and 500 ppm. Detection was performed at an absorbance of 280 nm. The utilization of the retention times comparison of the phenolic acid standards was instrumental in the identification of individual phenolic acids present within the samples and quantified based on the respective peak areas.

### 2.5. 3T3-L1 Cell Differentiation

For cell culture experiments, 3T3-L1 pre-adipocytes (No. 60159; BCRC, Hsinchu, Taiwan) were utilized. The cells were cultivated in 6-well plates under 37 °C with 5% CO_2_ conditions, using DMEM medium enriched with a 10% concentration of bovine calf serum. The differentiation process of 3T3-L1 pre-adipocytes was initiated through adding adipogenic agents (1 µM insulin, 0.5 mM IBMX, and 1 µM dexamethasone). Following eight days of differentiation, the differentiation medium was substituted with standard medium, and the medium was replaced every 48 h to ensure sustained cell growth.

### 2.6. Evaluation of Lipid Accumulation in 3T3-L1 Adipocytes

Adipogenic differentiation was assessed by visualizing and quantifying neutral lipid storage via Oil Red O (ORO) staining [27]. Mature 3T3-L1 cells were exposed to the indicated EEHT dosages (0, 100, or 250 μg/mL) for a 48 h window. Post-treatment, the culture medium was discarded, and the cells were rinsed with PBS buffer before being stabilized in a 10% formaldehyde solution for 20 min. Following fixation, the cellular monolayer underwent dehydration using 100% propylene glycol for 3 min and was subsequently washed with 60% propylene glycol. The specimens were then saturated with ORO reagent for 60 min and subsequently dried in a 37 °C environment. Microscopic documentation was performed at 200× magnification using an inverted microscope (Olympus, Osaka, Japan), with droplet analysis conducted via ImagePro Plus software, version 7.0 (Media Cybernetics, Inc., Rockville, MD, USA). For quantitative measurement, the ORO dye was eluted using pure isopropanol, and the absorbance of the resulting solution was recorded at 520 nm utilizing a FLUOstar microplate reader (BMG Labtechnologies, Offenburg, Germany). The percentage of lipid accumulation was determined by comparing the absorbance of the treated samples to that of the control group.

### 2.7. Intracellular Triglyceride (TG) Quantification

Mature 3T3-L1 adipocytes were mixed with 0, 100, and 250 μg/mL of either EEHT or individual phenolic acid standards for a 48 h window. A cold PBS-based lysis buffer with 1% Triton X-100 was utilized to resuspend and homogenize the detached cells. To obtain a clear supernatant, the resulting homogenate was subjected to centrifugation at 10,000 rpm for 10 min at 4 °C to remove cellular debris before further analysis. The contents of total intracellular triglycerides (TG) in the supernatant were subsequently evaluated using a commercial diagnostic suite (DiaSys Diagnostic Systems, Holzheim, Germany) according to the manufacturer’s guidelines. To account for variations in cell density, all TG measurements were normalized against the total cellular protein concentration, which was estimated using a standard Bradford protein assay. The degree of inhibition was determined by normalizing the TG levels against the untreated control (defined as 0% inhibition), expressed as a percentage reduction in lipid accumulation.

### 2.8. Statistical Analysis

The experimental procedure was conducted in triplicate. All data were examined using the Statistical Product and Service Solutions (SPSS) software, version 24.0 (IBM Corp., Armonk, NY, USA). Duncan’s new multiple-range test was used to examine the variances among the groups. PROC ANOVA was used to analyze variance. Furthermore, a Student’s *t*-test was employed to determine the statistical significance of the differences between the intervention doses in the cell experiments. A *p*-value < 0.05 indicates a verifiable difference between the groups.

## 3. Results

### 3.1. Total Polyphenolic Contents, Trolox Equivalent Antioxidant Capacities, and the Extraction Yields in Ethanol Extracts of Hsian-Tsao

The quantitative analysis of total phenolic content, antioxidant potential, and extraction yields across the various EEHT groups is summarized in Table 1. Among the tested solvents, the 60% ethanol extract (60EEHT) achieved the most substantial extraction yield, recorded at 21.23 ± 0.18% (*p* < 0.05). Notably, the 40% ethanol extract (40EEHT) demonstrated a superior profile, yielding the highest concentration of total polyphenols (155.33 ± 0.74 mg/g extract) alongside the most potent radical-scavenging activity (1.71 ± 0.07 μmol/g extract) (*p* < 0.05).

### 3.2. The Contents of Phenolic Acids in Ethanol Extracts of Hsian-Tsao

As detailed in Table 2, the distribution of specific phenolic acids varied significantly across the ethanol gradients. While PCA was most prevalent in the aqueous extract (0EEHT) (0.53 ± 0.01 mg/g extract), CGA peaked in the 20% fraction (1.39 ± 0.00 mg/g extract), *p*-HBA was highest in 80EEHT (0.26 ± 0.01 mg/g extract), CA was highest in 40EEHT (19.61 ± 0.14 mg/g extract), VA was highest in 60EEHT (0.41 ± 0.00 mg/g extract), and *p*-CA was highest in 40EEHT (21.15 ± 2.92 mg/g extract). The 40EEHT group was characterized by the highest cumulative phenolic acid content (42.41 ± 3.05 mg/g extract) (*p* < 0.05). In this optimal fraction, *p*-coumaric acid (*p*-CA) and caffeic acid (CA) emerged as the dominant constituents, reaching concentrations of 19.61 ± 0.14 mg/g and 21.15 ± 2.92 mg/g, respectively (*p* < 0.05).

### 3.3. Effects of Hsian-Tsao Ethanol Extracts on Cell Number and Triglyceride Levels in 3T3-L1 Adipocytes

**Figure 1:**
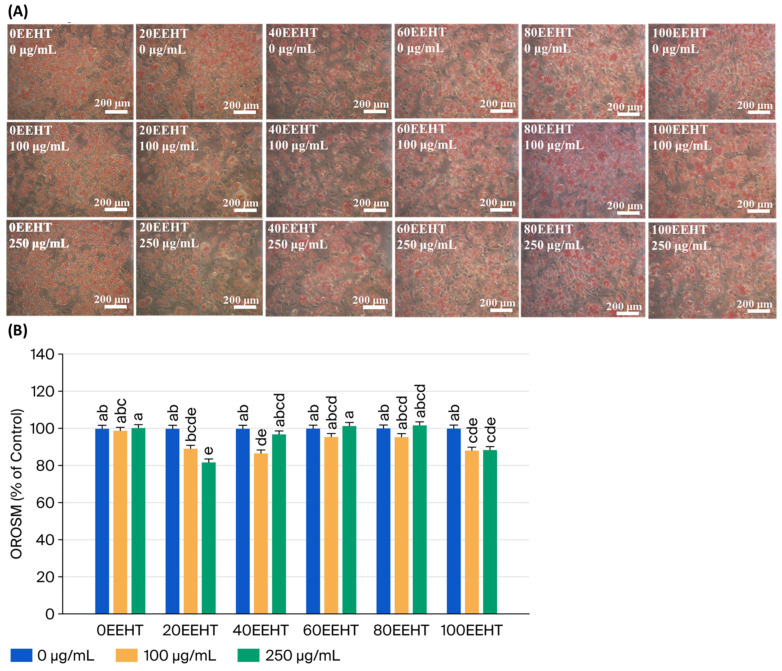
Effects of ethanol extracts of Hsian-tsao on cell counts in 3T3-L1 adipocytes. (**A**) The OROSM staining results of 3T3-L1 adipocytes with different EEHT ratios. Original magnification: 200×. (**B**) Cell counts (%) are expressed as 100% control. The cells were treated with 0, 100, and 250 μg/mL of 0, 20, 40, 60, 80, and 100% EEHT for 48 h. The reported values represent the means ± SEMs (*n* = 3), and the values with distinct letters were markedly different at *p* < 0.05; 0% ethanol extracts of Hsian-tsao (100% water extracts of Hsian-tsao), 0EEHT; 20% ethanol extracts of Hsian-tsao, 20EEHT; 40% ethanol extracts of Hsian-tsao, 40EEHT; 50% ethanol extracts of Hsian-tsao, 50EEHT; 60% ethanol extracts of Hsian-tsao, 60EEHT; 80% ethanol extracts of Hsian-tsao, 80EEHT; 100% ethanol extracts of Hsian-tsao, 100EEHT.

**Figure 2:**
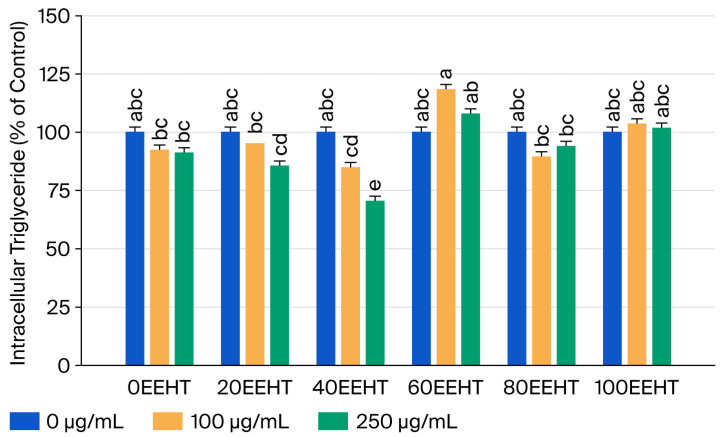
Effects of ethanol extracts of Hsian-tsao on the intracellular triglyceride contents of 3T3-L1 adipocytes. Cell counts (%) are expressed as 100% control. The 3T3-L1 adipose cells were exposed to 0, 100, and 250 μg/mL of 0–100% EEHT for 48 h. The reported values represent the means ± SEMs (*n* = 3), and the values with distinct letters were markedly different at *p* < 0.05; 0% ethanol extracts of Hsian-tsao (100% water extracts of Hsian-tsao), 0EEHT; 20% ethanol extracts of Hsian-tsao, 20EEHT; 40% ethanol extracts of Hsian-tsao, 40EEHT; 50% ethanol extracts of Hsian-tsao, 50EEHT; 60% ethanol extracts of Hsian-tsao, 60EEHT; 80% ethanol extracts of Hsian-tsao, 80EEHT; 100% ethanol extracts of Hsian-tsao, 100EEHT.

The influence of 0–100% EEHT on lipid droplet formation was visualized using Oil Red O (ORO) staining, as illustrated in Figure 1A. Our data show that the 3T3-L1 adipocyte cell counts were significantly decreased by treatment with 20EEHT (250 μg/mL), 40EEHT (100 μg/mL), and 100EEHT (100 and 250 μg/mL) (*p* < 0.05) (Figure 1B). The most pronounced reduction in intracellular triglyceride content was specifically observed with 250 μg/mL of 40EEHT (*p* < 0.05) (Figure 2). This suggests that at higher concentrations, 40EEHT primarily exerts an anti-adipogenic effect by suppressing lipid droplet formation rather than through generalized cytotoxicity.

### 3.4. Effects of EQR on the Short-Chain Fatty Acid Contents in HFD-Induced Obese Rats

**Figure 3:**
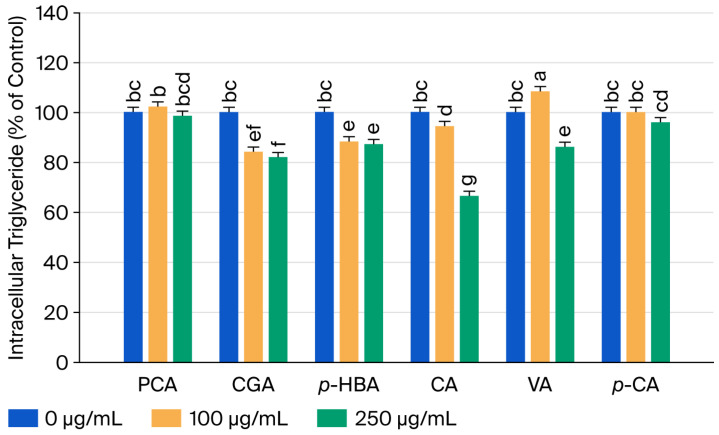
Effects of phenolic acid standards on the intracellular triglyceride content in 3T3-L1 adipocytes. Cell counts (%) are presented as 100% control. The 3T3-L1 adipose cells were combined with 0, 100, and 250 μg/mL of PCA, CGA, *p*-HBA, CA, VA, and *p*-CA for a 48 h window. The reported values represent the means ± SEMs (*n* = 3), and the values with distinct letters were markedly different at *p* < 0.05. Protocatechuic acid, PCA; chlorogenic acid, CGA; *p*-hydroxybenzoic acid, *p*-HBA; caffeic acid, CA; vanillic acid, VA; *p*-coumaric acid, *p*-CA.

To clarify the anti-obesity-related bioactive components in the EEHT, the 3T3-L1 adipose cells were treated with *p*-CA, CGA, CA, *p*-HBA, VA, and PCA, and then the intracellular triglyceride content was determined. Figure 3 showed that the intercellular triglyceride levels of 3T3-L1 adipocytes were significantly reduced by CGA (100 and 250 μg/mL), *p*-HBA (100 and 250 μg/mL), CA (100 and 250 μg/mL), and VA (250 μg/mL) (*p* < 0.05). Furthermore, 250 μg/mL of CA provided the optimal condition, significantly decreasing triglyceride contents in 3T3-L1 adipose cells by around 33.65%.

## 4. Discussion

Our study demonstrates a clear correlation between the ethanol extraction percentage, the resulting phenolic acid profile, and the biological efficacy of *Mesona procumbens* Hemsl. extracts. Specifically, 40EEHT exhibited the highest total polyphenol content (TPC) and superior antioxidant capacity (TEAC) among all tested fractions. While existing literature generally links high TPC to reduced oxidative stress and the prevention of chronic metabolic syndromes [28,29,30,31,32,33]. Our findings provide a more specific mechanistic link: the potent radical-scavenging activity of 40EEHT likely contributes to its anti-adipogenic effects. During the differentiation of 3T3-L1 adipocytes, the production of reactive oxygen species (ROS) acts as a critical signaling trigger for adipogenesis. Thus, the superior TEAC value of 40EEHT suggests that its bioactive components may suppress lipid accumulation by modulating the intracellular redox state.

The anti-obesity potential of 40EEHT was further validated by its ability to significantly reduce triglyceride content and lipid droplet accumulation in 3T3-L1 cells. Rather than a general effect observed in other botanical extracts like *Rosa rugosa* or peanut shells [34,35], the efficacy of 40EEHT is specifically attributable to its unique phenolic acid enrichment. Our HPLC analysis identified a comprehensive profile including PCA, CGA, *p*-HBA, CA, VA, and *p*-CA, with caffeic acid (CA) and *p*-coumaric acid (*p*-CA) being the most abundant. By individually testing these standards, we confirmed that CGA, *p*-HBA, and CA are the primary drivers of the observed anti-adipogenic activity.

Our findings significantly build upon and extend recent research concerning the anti-obesity effects of *M. procumbens*. Our research identifies the 40% ethanol extract as the most potent fraction due to its specific enrichment of phenolic acids. By systematically characterizing the phenolic acid profiles and testing individual compounds like caffeic acid and *p*-coumaric acid in 3T3-L1 cells. Recently, Cho et al. (2025) demonstrated that Hsian-tsao ethanol extracts effectively reduce body fat, serum lipids, and hepatic lipid accumulation in high-fat-diet-fed rats [36]. These findings further confirm the anti-lipid accumulation effects of Hsian-tsao ethanol extracts in vivo, paving the way for the future development of anti-obesity products.

In particular, the abundance of caffeic acid (CA) appears to play a pivotal role in the anti-obesity potential of 40EEHT. Mechanistically, our results suggest that CA exerts its effects by targeting multiple stages of lipid metabolism. While previous studies on CA derivatives like CAPE highlight the inhibition of mitotic clonal expansion (MCE) [37], our data regarding reduced triglyceride levels and ROS generation align with the understanding that CA suppresses lipogenesis and oxidative stress in differentiated adipocytes [38]. The efficacy of 40EEHT is likely since CA can modulate key metabolic regulators. For instance, CA is known to activate the AMPK pathway and inhibit ACC levels, thereby shifting the cellular balance toward mitochondrial β-oxidation and away from lipid synthesis [39,40]. Therefore, the anti-adipogenic effect of 40EEHT is not merely a collective result of “polyphenols,” but a targeted synergy driven by high concentrations of CA and its related phenolic acids. This data-driven conclusion suggests that the 40% ethanol extract optimizes the recovery of these specific bioactive compounds, making it the most potent fraction for lipid modulation. By enriching these specific phenolic acids through 40% ethanol extraction, 40EEHT provides an effective composition for impairing adipogenesis and promoting lipid catabolism in the 3T3-L1 model.

The scientific significance of this study lies in its systematic approach to identifying the specific phytochemical drivers behind the anti-obesity effects of *M. procumbens*. Although previous literature has reported the general bioactivity of Hsian-tsao extracts, most studies have focused on crude aqueous or generic ethanol extracts without optimizing the extraction parameters for specific bioactive enrichment. Our research bridges this gap by demonstrating that the 40EEHT possesses a superior concentration of total phenolic acids, specifically CA and *p*-CA, which significantly outweighs the potency of traditional aqueous extracts. Furthermore, by evaluating individual phenolic acid standards, this study provides a direct mechanistic link between 40EEHT’s chemical profile and its ability to suppress adipogenesis in 3T3-L1 cells. These findings offer a novel perspective on the standardization of Hsian-tsao-based functional ingredients, ensuring that future therapeutic applications are grounded in optimized phytochemical recovery rather than simple crude extraction.

Beyond its anti-adipogenic efficacy, the toxicological profile and safety of *M. procumbens* extracts warrant careful consideration for clinical translation. Traditionally, Hsian-tsao has been consumed as a food and beverage for centuries with a high threshold of safety. Our in vitro data align with this, showing no significant reduction in cell viability at therapeutic concentrations. However, the transition from traditional aqueous preparations to concentrated ethanol extracts (EEHT) may alter the dose–exposure relationship of bioactive phenolic acids. Previous animal studies have indicated that *M. procumbens* extracts exhibit low acute toxicity [12,36]. Nevertheless, high doses of isolated phenolic acids, such as caffeic acid, could theoretically interact with phase II detoxification enzymes or interfere with the pharmacokinetics of co-administered medications via cytochrome P450 modulation [41,42,43,44]. Furthermore, the concentration of specific compounds like CGA and CA in 40EEHT necessitates future in vivo dose-escalation studies to establish a No-Observed-Adverse-Effect Level (NOAEL). Addressing these safety parameters will be essential to ensure that the anti-obesity benefits of EEHT are not offset by unintended metabolic interactions or systemic side effects.

However, some limitations of the present study should be acknowledged. Adipogenesis is a sequential process involving early, middle, and terminal stages of differentiation. In this study, the EEHT treatments were conducted over a 48 h window, which primarily reflects the cumulative inhibitory effect on lipid droplet formation rather than a dynamic time-course progression. While this 48 h treatment is sufficient to demonstrate the anti-adipogenic potential of the extracts, further time-course experiments are required to precisely identify which specific stage of adipogenesis—such as mitotic clonal expansion or terminal differentiation—is most sensitive to EEHT components. Future research will focus on the temporal expression patterns of stage-specific markers to provide a more comprehensive mechanistic understanding.

While our results demonstrate that 40EEHT effectively suppresses lipid accumulation in a cellular model, these findings represent an initial screening of its anti-adipogenic activity. The observed effects suggest a potential for anti-obesity applications; however, further in vivo validation using animal models is warranted to confirm its systemic efficacy and safety.

## 5. Conclusions

According to our findings, 40EEHT is abundant in phenolic acids, including caffeic acid, protocatechuic acid, *p*-hydroxybenzoic acid, chlorogenic acid, *p*-coumaric acid, and vanillic acid. The polyphenolic constituents of 40EEHT, which possess high antioxidant capacity, contribute significantly to the targeted reduction in intracellular triglyceride content, demonstrating potential as a specific anti-adipogenic agent. Caffeic acid may be one of the major biofunctional components of 40EEHT. Overall, our data propose that EEHT has the potential to be developed into a beneficial supplement to combat obesity and related diseases.

## Figures and Tables

**Table 1:** The extraction yields, total polyphenol contents, and Trolox equivalent antioxidant capacities in ethanol extracts of Hsian-tsao.

Hsian-Tsao Extracts	Extraction Yields (%)	Total Polyphenol (mg/g Extract)	TEAC (μmol/g Extract)
0EEHT	16.65 ± 0.44 ^bc^	93.39 ± 1.00 ^f^	1.08 ± 0.02 ^c^
20EEHT	15.64 ± 1.08 ^c^	111.17 ± 0.32 ^d^	0.63 ± 0.06 ^d^
40EEHT	18.25 ± 1.44 ^b^	155.33 ± 0.74 ^a^	1.71 ± 0.07 ^a^
60EEHT	21.23 ± 0.18 ^a^	108.06 ± 0.21 ^e^	1.50 ± 0.08 ^b^
80EEHT	12.86 ± 1.04 ^d^	134.00 ± 0.75 ^b^	1.36 ± 0.07 ^b^
100EEHT	4.66 ± 0.28 ^e^	81.90 ± 0.75 ^g^	0.25 ± 0.01 ^e^

The data presented reflect the mean ± SEM (*n* = 3). Values with varying letters are considerably different (*p* < 0.05). Trolox equivalent antioxidant capacity, TEAC; 0% ethanol extracts of Hsian-tsao (100% water extracts of Hsian-tsao), 0EEHT; 20% ethanol extracts of Hsian-tsao, 20EEHT; 40% ethanol extracts of Hsian-tsao, 40EEHT; 50% ethanol extracts of Hsian-tsao, 50EEHT; 60% ethanol extracts of Hsian-tsao, 60EEHT; 80% ethanol extracts of Hsian-tsao, 80EEHT; 100% ethanol extracts of Hsian-tsao, 100EEHT.

**Table 2:** The phenolic acids contents in ethanol extracts of Hsian-tsao.

Hsian-Tsao Extracts	Phenolic Acids (mg/g Extract)						
	PCA	CGA	*p*-HBA	CA	VA	*p*-CA	Total
0EEHT	0.53 ± 0.01 ^a^	1.36 ± 0.00 ^bc^	0.19 ± 0.02 ^a^	2.06 ± 0.25 ^f^	0.04 ± 0.00 ^d^	1.33 ± 0.19 ^d^	5.52 ± 0.08 ^e^
20EEHT	0.13 ± 0.00 ^d^	1.39 ± 0.00 ^a^	0.21 ± 0.01 ^a^	6.79 ± 0.01 ^d^	0.10 ± 0.01 ^bcd^	2.07 ± 0.26 ^d^	10.68 ± 0.26 ^d^
40EEHT	0.16 ± 0.00 ^c^	1.36 ± 0.00 ^c^	0.07 ± 0.00 ^b^	19.61 ± 0.14 ^a^	0.07 ± 0.00 ^cd^	21.15 ± 2.92 ^a^	42.41 ± 3.05 ^a^
60EEHT	0.08 ± 0.00 ^e^	1.38 ± 0.00 ^ab^	0.03 ± 0.00 ^b^	12.18 ± 0.26 ^c^	0.41 ± 0.00 ^a^	11.18 ± 0.50 ^b^	25.25 ± 0.52 ^b^
80EEHT	0.08 ± 0.00 ^e^	1.37 ± 0.00 ^bc^	0.26 ± 0.01 ^a^	4.02 ± 0.15 ^e^	0.12 ± 0.01 ^c^	14.65 ± 0.71 ^b^	20.49 ± 0.87 ^c^
100EEHT	0.08 ± 0.00 ^e^	1.35 ± 0.01 ^c^	0.20 ± 0.09 ^a^	5.00 ± 0.92 ^e^	0.13 ± 0.05 ^b^	1.75 ± 0.33 ^d^	8.51 ± 0.72 ^de^

The data presented reflect the mean ± SEM (*n* = 3). Values with varying letters are considerably different (*p* < 0.05). Protocatechuic acid, PCA; chlorogenic acid, CGA; *p*-hydroxybenzoic acid, *p*-HBA; caffeic acid, CA; vanillic acid, VA; *p*-coumaric acid, *p*-CA; 0% ethanol extracts of Hsian-tsao (100% water extracts of Hsian-tsao), 0EEHT; 20% ethanol extracts of Hsian-tsao, 20EEHT; 40% ethanol extracts of Hsian-tsao, 40EEHT; 50% ethanol extracts of Hsian-tsao, 50EEHT; 60% ethanol extracts of Hsian-tsao, 60EEHT; 80% ethanol extracts of Hsian-tsao, 80EEHT; 100% ethanol extracts of Hsian-tsao, 100EEHT.

## Data Availability

The original contributions presented in this study are included in the article. Further inquiries can be directed to the corresponding author.

## Abbreviations

The following abbreviations are used in this manuscript:

ACC	Acetyl-CoA carboxylase
AMPK	5′ AMP-activated protein kinase
CA	Caffeic acid
CGA	Chlorogenic acid
DEX	Dexamethasone
DMSO	Dimethyl sulfoxide
EEHT	Ethanol Extracts of Hsian-tsao (*Mesona procumbens* Hemsl.)
HPLC	High-performance liquid chromatography
IBMX	3-isobutyl-1-methylxanthine
NOAEL	Observed-Adverse-Effect Level
ORO	Oil Red O
*p*-CA	*p*-Coumaric acid
PCA	Protocatechuic acid
*p*-HBA	*p*-Hydroxybenzoic acid
ROS	Reactive oxygen species
TEAC	Trolox Equivalent Antioxidant Capacity
TG	Triglyceride
TPC	Total Polyphenol Content
VA	Vanillic acid
